# A Centre for the Diagnosis and Treatment of Tuberculosis (CDT) in a resource-limited setting: a dragnet for patients with heart disease?

**DOI:** 10.1186/2049-3258-72-26

**Published:** 2014-08-04

**Authors:** Ahmadou M Jingi, Jean Jacques N Noubiap, Edvine Wawo Yonta, Philippe Kamdem, Joël Marie Obama, Samuel Kingue

**Affiliations:** 1 Department of Internal Medicine and Specialties, Faculty of Medicine and Biomedical Sciences, University of Yaoundé I, Yaoundé, Cameroon; 2 Internal Medicine Unit, Edéa Regional Hospital, PO Box 100, Edéa, Cameroon; 3 Department of Internal Medicine, Yaoundé Teaching Hospital, Yaoundé, Cameroon; 4 Centre Médical de la Trinité, Bafoussam, Cameroon; 5 Department of Internal Medicine, Bertoua Regional Hospital, Bertoua, Cameroon; 6 Department of Internal Medicine, Yaoundé General Hospital, Yaoundé, Cameroon

**Keywords:** Cardiac disease, Center for the diagnosis and treatment of tuberculosis, Sub-Saharan Africa, Cameroon

## Abstract

**Background:**

Cardiovascular disease is a growing public health problem in sub-Saharan Africa. Cough and dyspnea are symptoms of both lung diseases and heart failure. This study aimed at determining the contribution of cardiac diseases versus pulmonary diseases in the etiological profile of patients presenting with cough and dyspnea in a Center for the Diagnosis and Treatment of Tuberculosis (CDT), in a semi-rural area in Cameroon.

**Methods:**

This is a cross-sectional analysis of data from patients aged 18 years or more who consulted for cough and or dyspnea between December 2009 and December 2010 at the CDT of Lafe-Baleng, Bafoussam, Cameroon.

**Results:**

A total of 1196 patients were received for various complaints during the study period; 348 (29.1%) of them presented with cough and or dyspnea, and were included in the study. 186 patients (53.4%; 95% CI: 48.2-58.6) had a pure cardiac disease, while 122 patients (35.1%; 95% CI: 30.2-40.2) had a pulmonary disease. The prevalence of hypertension was 50.9%, and hypertensive heart disease was the most frequent cardiac disease with a prevalence rate of 37.6%. Heart failure was diagnosed in 222 patients, representing 63.8% (95% CI: 58.9-68.9) of patients with cough and or dyspnea, and 18.6% (95% CI: 16.5-21.0) of all the patients received at the CDT of Lafe-Baleng during the study period. Compared to patients with a pulmonary disease, patients with cardiac disease were older (*p* < 0.001) and more likely to present with dyspnea (*p* < 0.001) and to have hypertension (*p* < 0.001).

**Conclusion:**

We found a high prevalence of heart failure in this Centre for the Diagnosis and Treatment of Tuberculosis thus, a veritable dragnet for patients with heart disease. Our findings emphasize the urgent need to increase the access to cardiovascular care and to continuously raise the awareness of the communities on cardiovascular diseases in Cameroon.

## Background

Heart failure is a growing public health problem in sub-Saharan African countries [1,2]. In these countries, heart failure is mainly due to hypertension, valvular heart disease, and cardiomyopathy, with hypertension having the highest disease burden [3-8]. Cough and dyspnea are common symptoms in heart failure and lung diseases, with dyspnea quasi constant in heart failure [6]. Cough and dyspnea are main complaints of patients presenting in a clinic for lung diseases, especially in a Centre for the Diagnosis and Treatment of Tuberculosis (CDT). A CDT primarily focuses on lungs and usually does not provide specialized cardiovascular care. This main focus on lungs may lead to delay in the diagnosis of cardiac diseases among some patients presenting with cough and dyspnea in a CDT, causing harm to these patients and leading to unnecessary costs to the health services.

The CDT of Lafe-Baleng is a semi-urban health facility and a pioneer center for diagnosis and treatment of tuberculosis in the West region of Cameroon. As implemented by the national program to fight against tuberculosis, diagnosis tests for tuberculosis in the CDT of Lafe-Baleng are subsidized and the treatment free [9]. Individuals in the community who have a chronic cough and or dyspnea are considered suspects of lung infections, especially tuberculosis, and are therefore likely to consult in this center. This study was conducted to determine the contribution of cardiac diseases versus pulmonary diseases in the etiological profile of patients presenting with cough and dyspnea in this setting.

## Methods

### Study setting, participants and data collection

The study was conducted in the CDT of Lafe-Baleng which is located in Bafoussam, the capital of the West Region of Cameroon. This region has a population estimated at 1,800,000 inhabitants [10], and is characterized by a very limited access to effective interventions for prevention, diagnosis and treatment of cardiovascular diseases. At the time the study was conducted, there were only two cardiologic clinics in the entire region. The CDT of Lafe-Baleng has as main goal the diagnosis and treatment of tuberculosis, but other affections are also diagnosed and managed beside tuberculosis. No specialized cardiologic care is available in the center.

Patients received at the CDT of Lafe-Baleng between December 2009 and December 2010, aged 18 years and above, who consulted for at least chronic cough and or dyspnea were included in the study. During the study period, all patients were evaluated by a physician who recorded their sociodemographic and clinical data, as well as results of complementary exams in a registry. Clinical evaluation included a detailed medical history and a careful physical examination. A sputum smear for Acid Fast Bacilli, a full blood count and a chest X-ray were realized for all the patients. Depending on the clinical orientation, complementary exams were performed including a 2D-cardiac Doppler ultrasound, an electrocardiogram (ECG), or blood chemistry and serology. All chests X-ray were interpreted by two radiologists. Trans-thoracic cardiac Doppler ultrasound and ECG were performed by the same cardiologist in a nearby private clinic.

For the purpose of this study, we retrieved in the medical files of eligible patients, data including age, sex, weight, blood pressure and final diagnosis after investigations. Diagnosis were classified as i) cardiac disease including hypertensive, valvular, rhythmic and ischemic heart disease, cardiomyopathies, pericarditis; ii) pulmonary disease including pulmonary tuberculosis, non-tuberculous pneumonia, chronic obstructive pulmonary disease, bronchitis, asthma, pleural tuberculosis; iii) cardio-pulmonary disease: cor pulmonale, or pneumonia coexisting with or decompensating an existing heart disease, iv) non cardiac-non pulmonary disease: anemia or Tietze syndrome. We used the Framingham clinical diagnostic criteria for heart failure [11]. All patients with a clinical diagnosis of heart failure, besides the systematic chest X-ray, underwent a 2D-cardiac Doppler ultrasound and an ECG to determine the most probable mechanism of heart failure. The criteria used for the echocardiographic diagnosis of the cardiac diseases reported in this study can be found in a previously published echocardiographic study [8].

### Data analysis

Data were coded, entered and analyzed using the Statistical Package for Social Science (SPSS) version 20.0 for Windows (SPSS, Chicago, Illinois, USA). We described continuous variables using means with standard deviations, and categorical variables using their frequencies and percentages. The t-test and the Chi-square test were used to compare quantitative and categorical variables respectively, and a *p* value less than 0.05 was considered statistically significant.

The study received a hospital local institutional ethical clearance.

## Results

A total of 1196 patients were received for various complaints in the CDT of Lafe-Baleng during the study period; 348 (29.1%) of them presented with cough and or dyspnea, and their records were considered for analysis. Two hundred and 2 (58%) were males; their ages ranged from 18 to 95 years with a mean of 58.4 years (SD = 16.9). The prevalence of hypertension was 50.9%. As shown in Table 1, pulmonary disease was seen in 122 patients (35.1%; 95% CI: 30.2-40.2), a cardiac disease in 186 patients (53.4%; 95% CI: 48.2-58.6), a cardio-pulmonary disease in 36 patients (10.3%; 95% CI: 7.6-14.0), and a non cardiac-non pulmonary disease in 4 patients (1.2%; 95% CI: 0.4-2.9). Hypertensive heart disease was the most frequent cardiac disease, affecting 131 patients (37.6%; 95% CI: 32.7-42.8). Heart failure was diagnosed in 222 patients, representing 63.8% (95% CI: 58.9-68.9) of patients with cough and or dyspnea, and 18.6% (95% CI: 16.5-21.0) of all the patients received at the CDT of Lafe-Baleng during the study period.

**Table 1:** Etiological profile of the study population

**Diagnosis**	**Frequencies (%) N = 348**	**95% CI**
**Cardiac disease**		
Hypertensive heart disease	131 (37.6)	32.7-42.8
Rheumatic heart disease	8 (2.3)	1.2-4.5
Ischemic heart disease	2 (0.6)	0.1-2.1
Arrhythmias	10 (2.9)	1.6-5.2
Cardiomyopathies	32 (9.2)	6.5-12.9
Pericarditis	3 (0.9)	0.2-2.7
**Total**	186 (53.4)	48.2-58.6
**Pulmonary disease**		
COPD	10 (2.9)	1.6-5.2
Non-tuberculous pneumonia	52 (14.9)	11.6-19.1
Pulmonary tuberculosis	17 (4.9)	3.1-7.7
Pleural tuberculosis	6 (1.7)	0.8-3.7
Bronchitis	36 (10.3)	7.6-14.0
Asthma	1 (0.3)	0.05-1.6
**Total**	122 (35.1)	30.2-40.2
**Cardio-pulmonary disease**		
Cor pulmonale	9 (2.6)	1.4-4.9
Pneumonia/Heart failure	27 (7.8)	5.4-11.1
**Total**	36 (10.3)	7.6-14.0
**Non cardiac and non-pulmonary disease**		
Anemia	1 (0.3)	0.05-1.6
Tietze syndrome	3 (0.9)	0.2-2.7
**Total**	4 (1.2)	0.4-2.9

COPD: Chronic Obstructive Pulmonary Disease.

As shown in Table 2, compared to patients with a pulmonary disease, those with a cardiac disease were older (*p* < 0.001), and were more likely to have systolic hypertension (*p* < 0.001) and or diastolic hypertension (*p* < 0.001). Cough was more frequent in patients with pulmonary disease (*p* < 0.001), while dyspnea was more frequent in patients with cardiac disease (*p* <0.001).

**Table 2:** Comparison between patients with cardiac disease and pulmonary disease

**Characteristic**	**Pulmonary disease (n = 122)**	**Cardiac disease (n = 186)**	**P-value**
	**n (%) or mean (SD)**		
**Age (years)**	50.9 (16.6)	63.5 (13.9)	<0.001
**Sex**			
**Male**	66 (54.1)	113 (60.8)	0.28
**Blood pressure**			
**Systolic BP**	118.1 (20.5)	150.1 (29.7)	<0.001
**Diastolic BP**	72.3 (14.6)	88.9 (19.7)	<0.001
**Systolic BP ≥ 140 mmHg**	14 (11.5)	118 (63.4)	<0.001
**Diastolic BP ≥ 90 mmHg**	13 (10.7)	95 (51.1)	<0.001
**Symptom**			
**Dyspnea**	4 (3.3)	61 (32.8)	<0.001
**Cough**	95 (77.9)	37 (19.9)	<0.001
**Dyspnea and cough**	23 (18.5)	88 (47.3)	<0.001
**Weight**	64.1 (14.6)	70.5 (19)	<0.001

## Discussion

Tuberculosis remains a major public health problem in sub-Saharan African countries like Cameroon [12]. In Cameroon, the incidence of all clinical forms of tuberculosis is about 25,000 new cases per year (National Tuberculosis Program [NTP] report no. 16, May 2011). As recommended by the World Health Organization, in Cameroon, the fight against Tuberculosis is organized by a national program which coordinates all the activities, and CDT at the community level provide subsidized diagnosis tests and free treatment [9]. People with respiratory symptoms like cough and dyspnea, and who may be affected either by respiratory disease or by heart disease are therefore likely to consult in these centers. This study aimed at determining the contribution of cardiac diseases versus pulmonary diseases in the etiological profile of patients presenting with cough and dyspnea in a center with main focus on tuberculosis, the CDT of Lafe-Baleng.

We found that 53.4% of patients presenting with cough and or dyspnea were affected by a cardiac disease. Besides, 10.3% of patients had both pulmonary and cardiac disease. Moreover, heart failure was diagnosed in 63.8% of these patients with cough and or dyspnea, and up to 18.6% of all the patients seen in the setting during the study period. These findings highlight the high burden of cardiac disease in our setting, and show that in a context of limited access to cardiovascular care, a CDT may be a dragnet for patients with heart disease. These findings also echo observations that have been made for several decades about the intersection between case-finding for tuberculosis and heart failure. For instance, between 1966 and 1968, Perry EH and Gordon CG observed that about 20% of all symptomatic patients received in the Addis Ababa Tuberculosis Center, Ethiopia, had a cardiac disease [13].

The spectrum of cardiac disease in our setting was similar to those previously reported in Cameroon and other Sub-Saharan African countries, with hypertensive heart disease having the highest burden [2-8]. In a recent study in an urban population in Cameroon, it has been found a high prevalence of hypertension with low awareness, treatment and control [14]. The high prevalence of hypertension (50.9%) that we found in our semi-rural setting is alarming and emphasize the need to increase the access to healthcare and continuously raise the awareness of the communities on cardiovascular diseases.

We found that compared to patients with a pulmonary disease, those with a cardiac disease were older (*p* < 0.001), presented with dyspnea (*p* < 0.001) or dyspnea and cough (*p* < 0.001), and were more likely to have hypertension (*p* < 0.001). Dyspnea is a quasi-constant symptom of heart failure, underlying the severity classification of heart failure [6,15]. Our findings suggest that in such a center with primary focus on lung, one should first think of the heart in the aging patients with dyspnea/cough and high blood pressure then, screen for a lung affection. The high prevalence of cardiac disease in our setting suggests to integrate cardiovascular care in CDT, especially in a semi-rural or rural area with low access to health care.

Our study has some limitations due to its retrospective nature, including the missing data, which are expected for a study conducted on data collected from patients’ files. This study does not provide detailed clinical description of the disease spectrum reported, and important risk factors of heart disease and co-morbidities were not assessed. Despite this shortcoming, our study provide data of clinical and public health relevance, which confirm the etiological profile of cardiac disease previously reported in our country, and draw attention on the prevalence of cardiac disease in a center for lung disease, especially in setting with low access to cardiovascular care.

## Conclusion

We found a high prevalence of heart failure in this Centre for the Diagnosis and Treatment of Tuberculosis thus, a veritable dragnet for patients with heart disease. These patients were older and more likely to present with dyspnea and to have hypertension compared to those with pure pulmonary disease. There is an urgent need to increase the access to cardiovascular care and to continuously raise the awareness of the communities on cardiovascular diseases in Cameroon.

## Competing interests

The authors declare that they have no competing interests. They have not benefited from any sponsorship and funding.

## Authors’ contributions

AMJ designed the study, collected and analyzed the data, critically reviewed and revised the manuscript. JJNN contributed in study design, analyzed the data, drafted and revised the manuscript. PK collected the data, critically reviewed and revised the manuscript. EWY, JMO and SK critically reviewed and revised the manuscript. All authors approved the final version of the manuscript.
